# Transcriptomic Analysis of Cardiomyocyte Extracellular Vesicles in Hypertrophic Cardiomyopathy Reveals Differential snoRNA Cargo

**DOI:** 10.1089/scd.2021.0202

**Published:** 2021-12-16

**Authors:** Victoria James, Zubair A. Nizamudeen, Daniel Lea, Tania Dottorini, Terri L. Holmes, Benjamin B. Johnson, Kenton P. Arkill, Chris Denning, James G.W. Smith

**Affiliations:** ^1^ School of Veterinary Medicine and Science, University of Nottingham, Nottingham, United Kingdom.; ^2^ Faculty of Medicine and Health Sciences, Norwich Medical School, University of East Anglia, Norwich, United Kingdom.; ^3^ School of Medicine, University of Nottingham, Nottingham, United Kingdom.; ^4^ Biodiscovery Institute, University of Nottingham, Nottingham, United Kingdom.

**Keywords:** cardiomyocytes, hypertrophic, pluripotent stem cells, extracellular vesicles

## Abstract

Hypertrophic cardiomyopathy (HCM) is characterized by increased left ventricular wall thickness that can lead to devastating conditions such as heart failure and sudden cardiac death. Despite extensive study, the mechanisms mediating many of the associated clinical manifestations remain unknown and human models are required. To address this, human-induced pluripotent stem cell (hiPSC) lines were generated from patients with a HCM-associated mutation (c.*ACTC1*^G301A^) and isogenic controls created by correcting the mutation using CRISPR/Cas9 gene editing technology. Cardiomyocytes (hiPSC-CMs) were differentiated from these hiPSCs and analyzed at baseline, and at increased contractile workload (2 Hz electrical stimulation). Released extracellular vesicles (EVs) were isolated and characterized after a 24-h culture period and transcriptomic analysis performed on both hiPSC-CMs and released EVs. Transcriptomic analysis of cellular mRNA showed the HCM mutation caused differential splicing within known HCM pathways, and disrupted metabolic pathways. Analysis at increasing contraction frequency showed further disruption of metabolic gene expression, with an additive effect in the HCM background. Intriguingly, we observed differences in snoRNA cargo within HCM released EVs that specifically altered when HCM hiPSC-CMs were subjected to increased workload. These snoRNAs were predicted to have roles in post-translational modifications and alternative splicing, processes differentially regulated in HCM. As such, the snoRNAs identified in this study may unveil mechanistic insight into unexplained HCM phenotypes and offer potential future use as HCM biomarkers or as targets in future RNA-targeting therapies.

## Introduction

Hypertrophic cardiomyopathy (HCM) is the most commonly inherited heart disease, with a prevalence now considered to be greater than 1 in 500 in the general population [1]. Clinically, HCM can be characterized by increased left ventricular wall thickness (>15 mm for adults) that is not solely explained by abnormal loading conditions [2]. Histologically, the hearts of HCM patients consist of enlarged cardiomyocytes, with alignment disarray, and the presence of interstitial fibrosis [3]. Genetically, HCM is an autosomal-dominant disorder mainly caused by mutations in genes encoding for contractile and structural proteins of the cardiac muscle sarcomere apparatus [4].

The penetrance of the HCM phenotype is variable, with mutation carriers presenting with symptoms that range from fully asymptomatic to fatal cardiac dysfunction [5]. Insights into the molecular mechanism behind HCM have advanced significantly, and it is now clear that HCM mutations can alter myofilament Ca^2+^ sensitivity leading to the abnormal contractility, energy depletion, and enhanced susceptibility to arrhythmia [6]. Yet the mechanisms leading to secondary HCM phenotypes, such as myocyte hypertrophy, myocardial disarray, and interstitial fibrosis, are not fully explained. It remains unclear how mutations in genes encoding sarcomeric proteins only expressed in cardiomyocytes, give rise to phenotypes that encompass multiple cardiac cell populations.

Extracellular vesicles (EVs) have been known to play roles in cell signaling for many years, but more recently, focus on smaller EVs (which include exosomes) has increased. Exosomes and smaller EVs usually between 30 and 150 nm in size, have the ability to transmit bioactive molecules between cells [7,8]. Blood contains vast amounts of exosomes released by many cell types, and the contribution of this complex signaling within the cardiovascular system is now increasingly investigated [9]. Circulating EVs have been found to contain a broad spectrum of small noncoding RNAs (snRNAs), including small nucleolar RNAs (snoRNAs) [10–12]. These snoRNAs are known to have a diverse range of functions, ranging from 2′-*O*-methylation and pseudouridylation of RNAs, through nucleolytic processing of rRNAs to the synthesis of telomeric DNA [13].

Studies have identified snoRNA involvement in cardiometabolic disease, including in lipid metabolism [14], diabetes mellitus [15], and doxorubicin cardiotoxicity [16]. In addition, snoRNAs have also been shown to regulate splicing in normal cardiac development [17] and can alter splicing in some congenital heart diseases (tetralogy of Fallot) [18]. Of interest, the knockdown of specific snoRNAs in mice models and rat cardiomyoblasts has been shown to reduce heart size and cell growth [19]. This suggests that snoRNAs may play a role in the regulation of healthy cardiac development; however, a regulatory role for snoRNAs in hypertrophy cardiomyopathy has not yet been reported.

The heart is a multicellular organ, with intercellular communication between cardiomyocytes, cardiac fibroblasts, vascular smooth muscle cells, and endothelial cells necessary to maintain normal cardiac function. EV signaling is involved in dynamic and two-way interactions that occur between cardiac cell populations, with it becoming clear that this signaling plays important roles in cardiovascular disease [20].

Cardiomyocyte exosomes are known to have a specific proteomic signature, with the presence of sarcomeric proteins (including cardiac actin) highlighting their myocardial origin [21]. In health, cardiomyocytes release cardioprotective exosomes, suppressing cardiac fibrosis [22]. Exercise has been shown to induce the secretion of cardiac exosomes containing anti-fibrotic miRNAs in rats [23], potentially preventing fibrosis accompanying exercise-induced cardiac hypertrophy. During damage, cardiomyocytes-derived and cardiac fibroblast-derived exosomes have been shown to induce hypertrophy and fibrosis [24,25]. However, it is still unclear how sarcomeric mutations change the EV profile and the potential signaling roles these play in HCM.

In this study, we aimed to investigate whether cardiomyocytes displayed abnormal EV signaling profiles during HCM. We further sort to determine whether this profile was altered during periods of increase work. To achieve this we utilized an isogenic pair of human-induced pluripotent stem cell-derived cardiomyocytes (hiPSC-CMs), with or without the c.*ACTC1*^G301A^ mutation. This model of HCM [26] was chosen for study as it had previously been shown to recapitulate many key disease phenotypes including abnormal contractility, Ca^2+^ sensitivity/handling, arrhythmogenesis, and hypertrophic brain natriuretic peptide signaling.

Using this isogenic model we were successfully able to isolate and perform transcriptomic analysis on EVs from wild type (WT) and HCM cardiomyocytes, both at baseline and increased contraction frequency. Comparisons between the HCM and WT EV transcripts identified multiple functional pathways affected by the presence of the disease mutation.

Transcriptomic analysis of cellular mRNA showed increasing contraction frequency had a large effect on metabolic gene expression, which was amplified in the HCM background. Analysis of small RNA (<200 nucleotides) showed HCM hiPSC-CMs altered their loading of specific snoRNAs into EVs, and responded to increased contraction frequency with a greater fold change in EV release rate, and changes in small RNA profile. The snoRNAs identified in HCM EVs at baseline and during increased work may have potential signaling roles in post-translational modifications (PTMs) and alternative splicing. These may in future be used as biomarkers or therapeutic targets.

## Materials and Methods

### hiPSC lines and culture

Isogenic hiPSC lines with or without the c.*ACTC1*^G301A^ mutation were generated as previously described in detail [26,27]. All cell culture experiments were performed in a type II Biological Safety Cabinet, and cells were incubated in a humidified incubator at 37°C and 5% CO_2_. hiPSCs were routinely maintained in E8 medium (Life Technologies) on 1:100 MT (Corning)-coated plastic ware (Nunc). Medium was changed every day and cells were passaged with TrypLE (Life Technologies) every 3 days, and used between passages 20 and 30.

### hiPSC-CM differentiation and electrical stimulation

Cardiomyocyte differentiation was performed by seeding hiPSCs on MT (Corning)-coated plastic ware at ∼20–40 thousand cells/cm^2^. Fresh E8 was added the next day and the preconditioning step of hiPSC was performed the day after, by adding an MT overlay (MT diluted 1:100 in StemPro™ 34-Serum Free Medium, SP34; Gibco) supplemented with 1 ng/mL BMP4 (R&D). Approximately 16 h later, medium was replaced by SP34 supplemented with 8 ng/mL Activin A (ActA; LifeTechnologies) and 10 ng/mL BMP4. Forty-eight hours later, medium was changed by RPMI supplemented with B27 without insulin (−INS; LifeTechnologies) and KY0211 (R&D) and XAV939 (R&D), both at 100 μM. These small molecules were added again 2 days later, in RPMI supplemented with B27 with insulin (+INS; LifeTechnologies) instead.

Thereafter, medium was changed every 2–3 days by fresh RPMI+B27+INS until day 15 of differentiation, when hiPSC-CM were replated, and kept in RPMI+B27+INS for ∼10 days when electrical stimulation and exosome isolation assays were performed. Electrical pacing of the hiPSC-CMs was performed with conditions of 1 V/2 Hz, impulse duration of 2 ms, using carbon electrodes (IonOptix) for 24 h.

### EV isolation and characterization

The conditioned media was centrifuged at 300 *g* for 10 min, 200 *g* for 10 min, and 10,000 *g* for 30 min at room temperature. The resulting supernatant was concentrated by Vivaspin-20 (100 MWCO) protein concentration column (Sigma Aldrich) following the manufacturer's directions to a total volume of 500 μL.

The concentrated supernatant was applied to a qEVoriginal (70 nm) size exclusion chromatography (SEC) column and EVs isolated according the manufacturer's direction. The resulting EVs were subject to western blot analysis of tetraspanin marker CD9, cytosolic marker Alix, and a Golgi marker negative control GM130. In brief, EVs and cells were resuspended in RIPA buffer supplemented with protease inhibitor cocktail (Promega). Protein concentration was determined by the Qubit protein assay (Invitrogen), and 20 μg of protein per EV sample and 10 μg per cell sample were separated by SDS-PAGE on 10% polyacrylamide gels (BioRad). Proteins were transferred to nitrocellulose membrane using the BioRad Transblot Turbo System.

Membranes were blocked for 1 h in 5% (w/v) bovine serum albumin (BSA) in TBST. Primary antibodies were incubated for 18 h at 4°C at a 1:1,000 dilution in 3% (w/v) BSA in TBST as follows: CD9 (D801A), Alix (3A49), and GM130 (D6B1) (Cell Signaling). Membranes were washed three times in TBST for 10 min, followed by a 60-min incubation with either HRP-conjugated anti-rabbit or anti-mouse secondary antibody (Cell Signaling) at room temperature. Membranes were washed three times in TBST for 10 min, followed by incubation with Clarity ECL (BioRad) and visualization using the BioRad GelDoc Go Imaging System.

The size and concentration of EVs was determined by electrophoresis and Brownian motion analysis using laser scattering microscopy and the resulting EVs were also characterized by nanosight and transmission electron microscopy and the Nanosight LM10 (Malvern Panalytical).

Finally, EVs were visualized by transmission electron microscopy for ultrastructural characterization. EV SEC elutes were fixed at a 1:1 ratio in 4% (v/v) paraformaldehyde (PFA) in cacodylate buffer for 18 h. The fixed EVs were seeded onto 0.1% poly-l-lysine-treated carbon/formvar slot grids (EM resolution) and embedded for 30 min at room temperature. The grids were washed twice with distilled water and stained with 1% aqueous uranyl acetate and left to air dry. Transmission electron microscopy was carried out (T 100KEV; FEI Tecnai Biotwin-12) with a side mount megaview III camera.

### RNA isolation and bioinformatics analysis

RNA was extracted using the miRNeasy miRNA isolation mini kit (Qiagen). RNA was prepared for sequencing using the NEBNext library preparation kits for the cell lines and the NEBNext Low Input library preparation kit for the EV samples (New England Biolabs). The RNAseq was carried out at the Barts Genome Centre (Queen Mary University of London) using the NextSeq 500 High Output Run (150 cycles) for all samples. The sequencing data were analyzed using a pipeline consisting of FastQC for quality control, Skewer [28] for read trimming, HiSat2 [29] for read alignment (to GRch38 build), StringTie [30] for transcript quantification, and DESeq2 [31] and Snakemake [32] to calculate differential expression. Pathway analysis was conducted using PANTHER V16.0 Classification System (www.pantherdb.org). Volcano plots were generated using VolcaNoseR.

## Results

### Transcriptomic analysis of HCM hiPSC-CM model

Skin-punch biopsies were previously obtained from a 48-year old male patient carrying the c.*ACTC1*^G301A^ HCM-causing mutation and reprogrammed to hiPSCs under ethical consent by labs in Nottingham (REC ref:09/H0408/74) [26]. Isogenic WT controls were generated for these hiPSCs using a footprint-free PiggyBac-based CRISPR/Cas9 strategy [27]. Differentiation of these hiPSC lines to high purity (>90% α-actinin^+^) cardiomyocytes (hiPSC-CMs) produced an in vitro model in which the mutant cells displayed characteristic HCM phenotypes when compared with the isogenic control line [26,33]. A summary of these HCM phenotypes is given in Supplementary Fig. S1, with HCM hiPSC-CMs displaying increased arrhythmias, hypercontractility, calcium dysregulation, and inefficient energy metabolism.

To determine if the HCM phenotypes seen within the cellular model could be analyzed at an expression level, transcriptomic analysis was performed on the cellular mRNA. Here we found 127 genes were dysregulated between the WT and HCM hiPSC-CMs (Supplementary Fig. S1b). Of these, 70% of genes were associated with controlling metabolic processes, indicating the background presence of the disease mutation leads to global alterations in metabolic processes, consistent with the observed energy depletion phenotype.

**FIG. 1.**
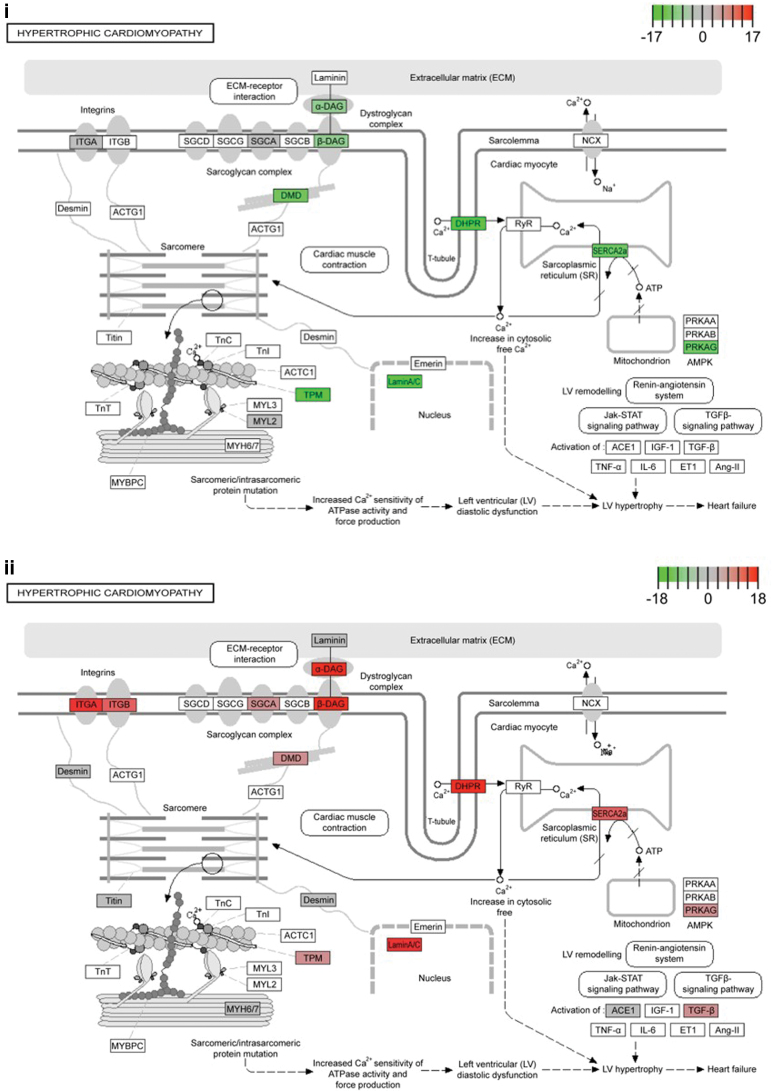
Transcriptomic analysis of hiPSC-CMs identified altered genes within the HCM pathway. **(i)** Genes with transcripts of increased expression are given in *green*. **(ii)** Genes with transcripts of decreased expression are given in *red*. hiPSC-CM, human-induced pluripotent stem cell cardiomyocyte; HCM, hypertrophic cardiomyopathy. Color images are available online.

Of importance, pathway analysis confirmed the disease mutation had led to disruption of known HCM pathway genes (Fig. 1). Of interest, multiple genes within this pathway were shown to have isoforms with increased and decreased expression, indicating the HCM mutant lines may alter the expression of alternative spliced variants.

Together this analysis confirmed the HCM phenotypes seen within the cellular model corresponded with altered transcript expression, and this could be identified through bioinformatics analysis. The presence of the c.*ACTC1*^G301A^ mutation had altered genes within the defined HCM pathway (KEGG pathway hsa05410), with differential isoform expression, as well as the dysregulation of global metabolic processes.

### Altered EV signaling in HCM

Following the confirmation of characteristic HCM cellular gene expression in the mutant hiPSC-CMs, we next sort to determine whether there was altered transcript cargo within EVs released by HCM hiPSC-CM. EVs released into culture medium by hiPSC-CMs were isolated after 24 h using SEC. These physical properties were characterized using nanoparticle tracking analysis (NTA), transmission electron microscopy, and western blots to determine the quality of the EV isolations and that the profiles corresponded to exosomes and smaller EVs (Supplementary Fig. S2).

**FIG. 2.**
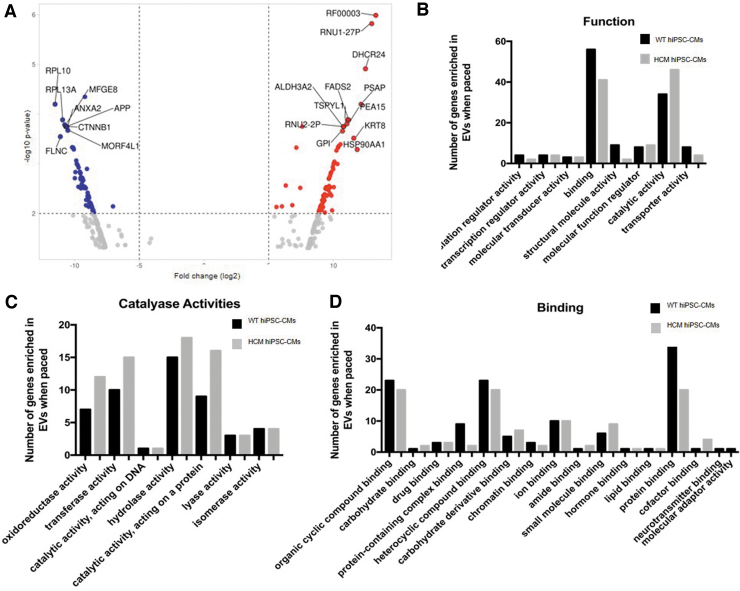
Transcriptomic analysis of HCM hiPSC-CM EVs identified differential cellular expression between WT and HCM hiPSC-CM. **(A)** Volcano plot analysis of the differential expression of total RNAs between WT and HCM hiPSC-CM EVs. A comparison of fold change (log2) and significance (−log10 *P*-value). RNAs significantly increased in abundance in HCM hiPSC-CM EVs (*red*) and in WT hiPSC-CM EVs (*blue*). Significance is determined as adj-*p* < 0.05. **(B)** Biological process enrichment analysis conducted using Panther to determine the number of genes per function pathway present in EVS produced from either the WT and HCM hiPSC-CMs. **(C)** Detailed analysis of the catalyase activity pathways and **(D)** detailed analysis of the binding pathways accounting for the number of mRNAs found in WT and HCM hiPSC-CM EVs per pathway. Specific genes and their functions are detailed in the Supplementary Figures S1–S5. EV, extracellular vesicle; WT, wild type. Color images are available online.

The isolated EVs were processed for transcriptomic analysis (Fig. 2). Biological process enrichment analysis was conducted using Panther to determine the number of genes per function pathway present in EVs produced from either the WT and HCM hiPSC-CMs (Fig. 2B). Of the functional pathways identified, catalytic activity and binding were the two pathways with the highest numbers of transcripts identified. Binding molecules have direct interactions with other molecules, and molecules with catalytic activity are involved in enzymatic reactions following the required binding. It was observed that EVs from HCM hiPSC-CMs had an increase in the number of transcripts in the catalytic activity pathway, and a decrease in the number of transcripts in the binding pathway compared with EVs from WT hiPSC-CMs.

Detailed analysis of the catalase activity pathways identified “activity acting on a protein” as the pathway with the greatest increase within the EVs from hiPSC-CMs compared with EVs from WT hiPSC-CMs (Fig. 2C). Similarly, the detailed analysis of the binding pathways identified “activity acting on a protein” as the pathway with the greatest decrease within the EVs from hiPSC-CMs compared with EVs from WT hiPSC-CMs (Fig. 2D).

The specific genes and their functions identified in these two pathways are detailed in Supplementary Figures. S3 and S4. Of note, genes associated with aspects of calcium signaling such as *CAMK2D*, *ATP2B4*, and *CAPN1*, and cardiac-specific structural genes such as *TNNT2*, *TPM1*, *TPM3*, and *TPM4*, were found within these pathways. Small RNA cargo analysis also identified differential levels of snoRNAs between WT and HCM hiPSC-CMs, and these were predicted to have roles in PTMs and alternative splicing (Supplementary Fig. S5). In total, 12 snoRNAs were identified including 10 SNORDs (SNORD6, SNOTRD116-23, SNORD116-25, SNORD116-29, SNORD18A, SNORD42A, SNORD43, SNORD58C, SNORD60, and SNORD 101) and 2 SNORAs (SNORA3B and SNORA20).

**FIG. 3.**
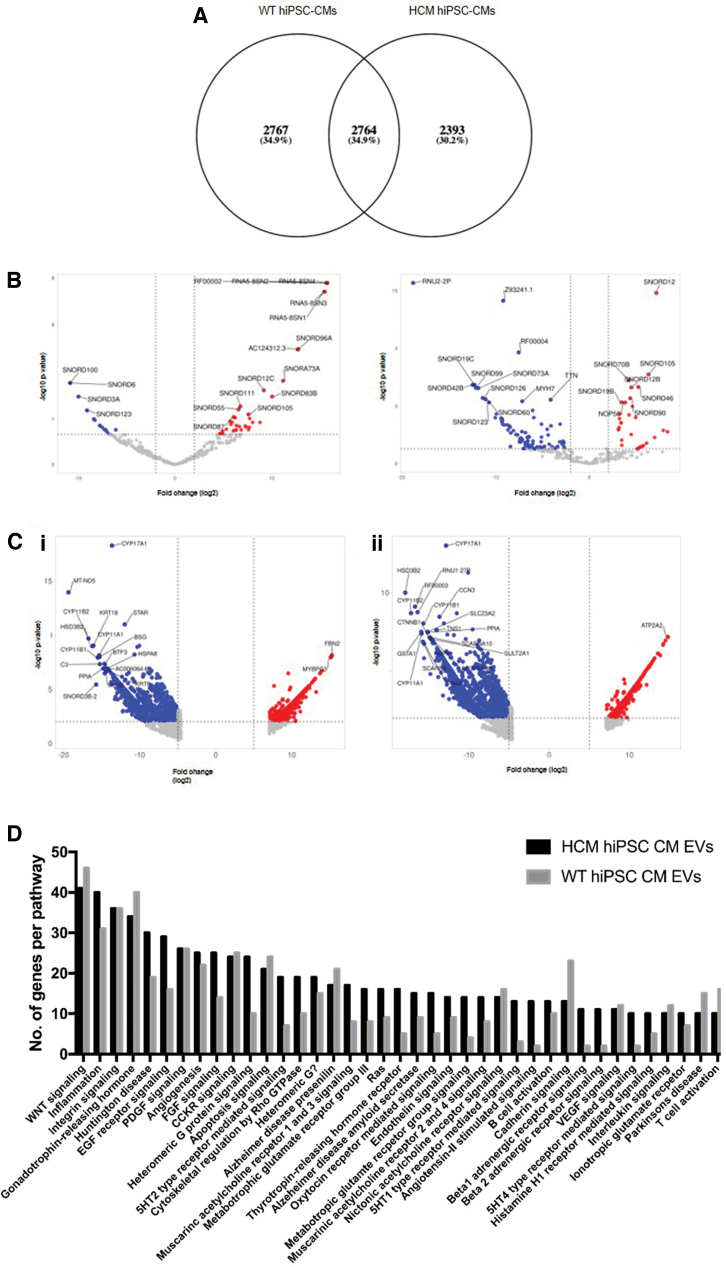
Comparison of RNAs enriched in EVs compared with their parental cell lines identified both mutation dependent and independent changes. **(A)** Of the mRNAs enriched in EVs, 34.9% are independent of the disease mutation, whereas 65.1% are mutation dependent. **(B)** Volcano plot analysis of the enriched exosome small RNA and **(C)** enriched exosome total mRNA of fold change (log2) and significance (−log10 *P*-value). RNAs significantly increased in abundance in cells compared with EVs (*red*) and EVs compared with cells (*blue*). **(i)** Results of comparing WT hiPSC-CMs cells with WT hiPSC-CMs EVs. **(ii)** Comparison of HCM hiPSC-CMs cells with HCM hiPSC-CMs EVs. Significance is determined as adj-*p* < 0.05. Data limited to the top 5000 hits demonstrating greatest difference in expression. **(D)** Pathway enrichment analysis of the mRNAs found to be unique to either WT hiPSC-CMs EVs or HCM hiPSC-CMs EVs. Color images are available online.

Following the comparison of transcripts within the HCM hiPSC-CM-released EVs with WT hiPSC-CM-released EVs, we subsequently compared the transcripts enriched within the EVs with their parental cell lines. Here we found that 34.9% of enriched transcripts were independent of the disease mutation, whereas 65.1% were mutation dependent (Fig. 3A). Comparison of the enriched EV small RNA (Fig. 3B) and enriched EV total mRNA (Fig. 3C) with the corresponding parental cell line identified common transcripts between the WT and HCM EVs, including many snoRNAs (such as enriched SNORD123 and depleted SNORD105) and cytochromes P450 genes (CYPs), that were enriched within EVs independent of the disease mutation (such as CYP11B1 and CYP17A1).

Pathway enrichment analysis of the mRNAs found to be mutation dependent and therefore unique to either WT hiPSC-CMs or HCM hiPSC-CMs EVs identified many potential functions of the enriched mRNAs within multiple pathways (Fig. 3D). Of note, HCM hiPSC-CM EVs showed an increased presence in unique transcripts associated with EGF and FGF signaling, 5-HT receptor-mediated signaling, angiotensin receptor signaling, beta 1 and beta 2 adrenergic signaling, and G protein receptor signaling. HCM hiPSC-CM EVs also showed a reduction in the presence of unique transcripts associated with cadherin signaling.

Together these data showed we were able to isolate and perform transcriptomic analysis on the cargo of HCM and WT hiPSC-CMs EVs. Comparisons between the HCM and WT EVs transcripts identified “catalytic activity on a protein” and “protein binding” as the two functional pathways most affected by the presence of the disease mutation. Comparisons of either the HCM or the WT EVs and their parental cell lines showed that the majority (around two thirds) of enriched transcripts were dependent on disease mutation. Of these, pathway enrichment analysis of the mRNAs found to be mutation dependent identified many signaling pathways that may be influenced by the altered HCM EV transcripts. Small RNA analysis showed the HCM hiPSC-CMs altered their loading of specific snoRNAs into EVS, potentially altering splicing and PTMs in recipient cells.

### Altered response to increased cardiac contraction in HCM

**FIG. 4.**
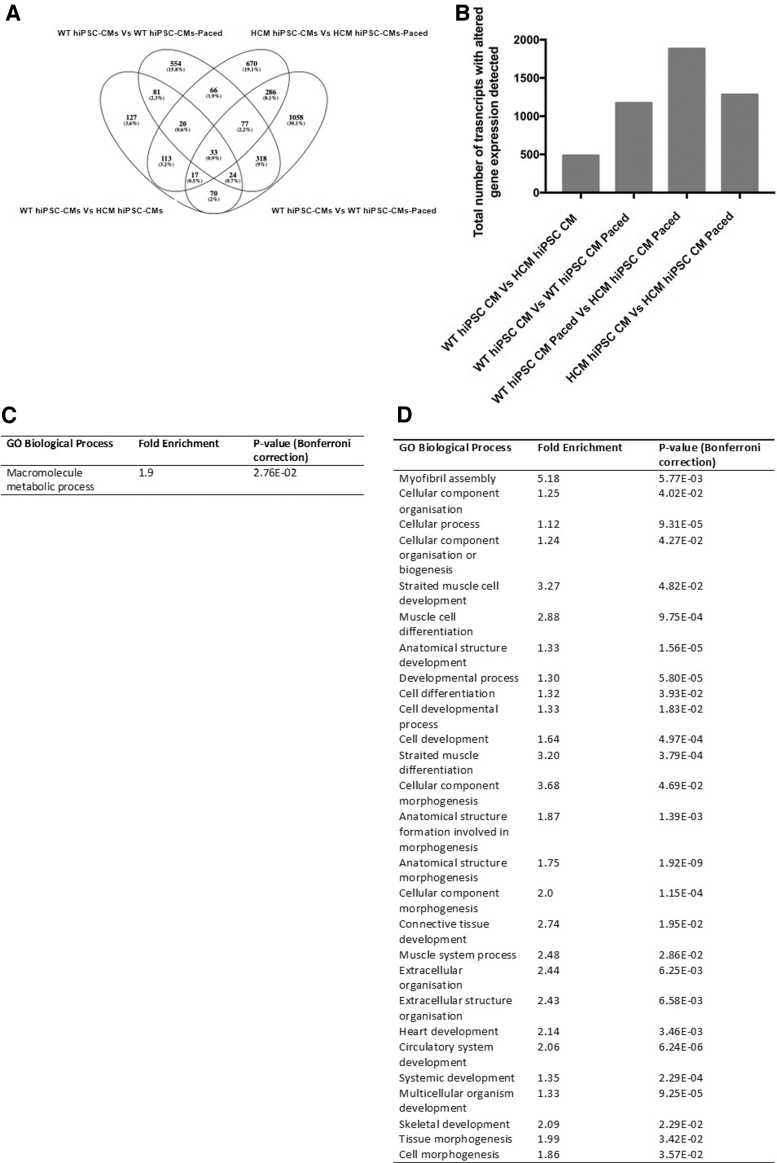
Altered cellular gene expression detected as a result of the presence of disease mutation and/or pacing as the stimulus. **(A)** Breakdown of comparisons of transcripts with altered abundance detected in hiPSC-CMs as a result of the presence of the disease mutation and/or pacing stimulus. **(B)** Summary of the total number of transcripts with altered abundance as a result of either the presence of the mutation and/or pacing stimulus. **(C)** Biological processes regulated by changes in transcript expression mediated by pacing stimulus alone. **(D)** Biological processes regulated by changes in transcript expression altered during pacing stimulus by the presence of the disease mutation.

Many of the most damaging phenotypes associated with HCM only present during periods of increased stress and cardiac work. We next sort to determine if we could identify differential responses between the WT and HCM hiPSC-CMs when subjected to an increased contraction frequency of 2 Hz, compared with baseline 1 Hz. Transcriptomic analysis of cellular mRNA showed that electrically stimulating the hiPSC-CMs at 2 Hz, simulating a moderate–vigorous physical intensity, had a large effect on gene expression (Fig. 4A). The presence of both the mutation and 2 Hz pacing appeared to create an additive effect, suggesting each factor alters the abundance of independent sets of genes (Fig. 4B).

Of the 66 genes that demonstrated altered expression as a result of 2 Hz pacing stimulation regardless of the disease mutation, 40 were identified within the macromolecule metabolic process (including the MAP kinases, *MAPK3* and *MAPK12*), indicating altered metabolic expression as a result of the increased contraction frequency (Fig. 4C). The additive effect of the 2 Hz pacing in the presence of the disease mutation was observed by the altered expression of 1,058 genes independent of these two factors.

GO enrichment analysis of these genes identified that many potential biological processes were altered by the combined effect of these two factors (Fig. 4D). These included genes associated with processes such as biogenesis and morphogenesis (including contractile myosins *MYL2*, *MYH3*, and *MYH6*), cardiac and circulatory development (including the cardiac transcription factors *TBX1* and *TBX18*), and cellular communication and signaling (including *BMP2* and *NOTCH2*).

**FIG. 5.**
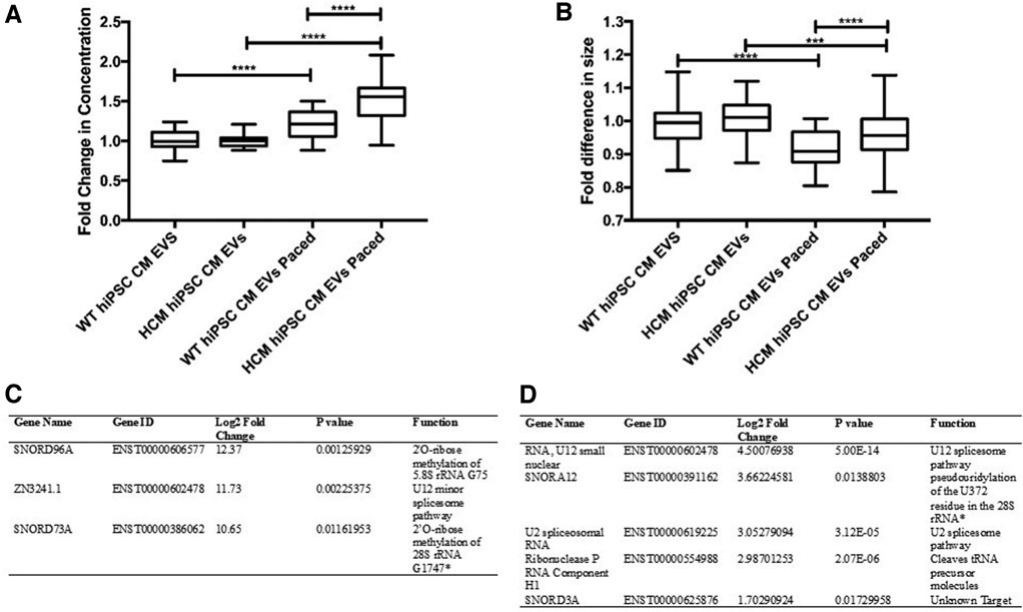
Altered EVs release and small RNA cargo detected as a result of the presence of disease mutation and/or pacing as the stimulus. **(A)** Quantification of EV release following pacing frequency stimulus. **(B)** Quantification of EV size following pacing frequency stimulus. **(C)** Small RNA with increased abundance (>2fc) in HCM hiPSC-CM EVs compared with WT hiPSC-CM when stimulated to pace. **(D)** Small RNA with increased abundance (>2fc) in HCM hiPSC-CM EVs when paced compared with HCM hiPSC-CM EVs when unstimulated. **(A, B)** One-way analysis of variance performed with correction (Turkey). *P ≤ 0.05, ***P ≤ 0.001, ****P ≤ 0.0001. (*n* = 3 biological replicates) per assay **(C, D)** Bonferroni correction.

Following the cellular comparisons, EVs released by both WT and HCM hiPSC-CMs during 2 Hz pacing were isolated to determine if RNA changes could also be detected within signaling vesicles. Of interest, when EV release was quantified through the use of NTA, it was observed that release rates differed between WT and HCM hiPSC-CMs. HCM hiPSC-CMs increased the release of EVs 1.5-fold during 2 Hz stimulation, compared with WT hiPSC-CM, which only responded with a 1.2-fold change (Fig. 5A). Size analysis showed modest changes in EV size distribution as a result of 2 Hz stimulation, indicating an increase in release corresponds with a decrease in EV size (Fig. 5B). A breakdown of this change in EV size distribution is given in Supplementary Fig. S2C.

Small RNA analysis of EV cargo following 2 Hz stimulus did not identify any changes in small RNA cargo in the EVs released from WT hiPSC-CMs when subjected to 2 Hz stimulation. However, comparing these WT EVs with the 2 Hz stimulated HCM hiPSC-CM EVs did identify differences in small RNA cargo (Fig. 5C). SNORD96A, SNORD73A, and ZN3241.1 were increased in 2 Hz stimulated HCM hiPSC-CM EVs compared with 2 Hz stimulated WT hiPSC-CM EVs. Furthermore, and in contrast with the WT line, it was shown that 2 Hz stimulation was sufficient to alter small RNA cargo in the HCM hiPSC-CM EVs, including snoRNAs, SNORD3A and SNORA12 (Fig. 5D). These small RNAs were predicted to have roles in PTMs and alternative splicing, indicating the HCM response to increased contraction frequency may further alter these processes.

Together, these data show that the response to an increased contraction frequency is altered in HCM hiPSC-CMs. Electrical stimulation alters metabolic pathways in WT hiPSC-CMs, but has an additive effect on many other biological pathways in HCM hiPSC-CMs. In addition, the cargo and release of EVs in response to the increased contraction frequency was significantly altered in HCM hiPSC-CMs. HCM hiPSC-CMs respond with a greater fold change in EV release rate compared with WT hiPSC-CMs and with a differential profile of loaded small RNAs, with potential signaling roles in PTMs and alternative splicing.

## Discussion

In this study we present transcriptomic analysis of an isogenic hiPSC-CM model of HCM. These data highlight some key cellular pathways altered in hypertrophic cardiomyocytes, as well as altered signaling during HCM pathogenesis. It should be noted that >1,400 mutations across >25 genes have been associated with HCM progression [34]. We have recently shown that different HCM-associated mutations can cause differences in disease signaling pathways, and as such a “one-size fits all” classification of HCM underestimates the complexity of the disease [6]. Future studies are therefore required to determine if the pathways reported for p.ACTC1-E99K mutant also contribute to alternative HCM mutants, especially those shown to have phenotypic differences such as p.β-MHC-R453C [33].

### Transcriptomic analysis of HCM cardiomyocytes

The variable penetrance of HCM and its manifold molecular mechanisms has hindered the development of efficient treatment options [6]. To reconcile the lack of consistency in the abnormalities that occur in HCM, a unifying model has been proposed in which HCM dysfunction is owing to an increased energy demand that results from inefficient sarcomeric ATP utilization [35]. This model is known as the “*energy depletion model*,” where increased energy demand compromises the capacity of the cardiomyocyte to maintain sufficient energy levels in subcellular compartments responsible for contraction and critical homeostatic functions (such as Ca^2+^ reuptake).

Isogenic hiPSC-CMs offer the only human model system that accounts for genetic background differences to unveil deeper mechanistic insights. Our isogenic hiPSC-CM model supports the theory that single sarcomeric mutations can lead to a global alteration of cardiomyocyte metabolic processes. We found that of the 127 genes that were dysregulated between the WT and HCM hiPSC-CMs (Fig. 1A), 70% were associated with controlling metabolic processes, indicating that this was the major cellular pathway altered, in agreement with the energy depletion model of HCM.

Pathway analysis confirmed the disease mutation had led to disruption of known HCM pathway genes (Fig. 1B). Of interest, we observed that for multiple genes within this pathway there was a switch in isoform expression, with specific isoforms increasing, whereas others within the same gene decreasing. This indicated the HCM mutant lines may differentially regulate alternative splicing.

The role of splicing in HCM is becoming an area of increased focus [36]. Proteomic analysis of primary tissue from the failing hearts of 16 HCM patients has recently revealed a common pattern of altered sarcomeric proteoforms compared with heart tissues from nonfailing donors [37]. Despite the complexity and heterogeneity of these HCM samples (including differences in disease-causing mutations, genetic background, age, and gender), this study observed consistent alterations in PTMs and changes in isoform expression within the HCM samples.

In addition, HCM mutations have been shown to cause aberrant splicing in an in vitro model, where minigenes were transfected into HEK293 cells [38]. However, it has been acknowledged that this is not an ideal system, as the mechanisms controlling splicing decisions are known to be influenced by chromatin structure and therefore are cell type specific. In this study, we used an isogenic hiPSC-CMs, accounting for differences in genetic backgrounds and physiological relevance of cell type-specific chromatin structure. In agreement with previous studies we found the presence of a sarcomeric HCM mutation altered splicing in multiple pathways, including the known HCM pathway.

### Transcriptomic analysis of HCM EVs

Although our understanding of the molecular mechanisms that underlie primary HCM phenotypes is advancing rapidly, the link between sarcomeric gene defects and “extended” HCM phenotypes (such as myocardial disarray, interstitial fibrosis, mitral valve abnormalities, and microvascular remodeling) remains less defined [39]. The presentation of these phenotypes indicates the involvement of other cell lineages and therefore a role in aberrant signaling between mutant cardiomyocytes and healthy neighboring population. The regulatory roles of cardiomyocyte-derived EVs through signaling to neighboring populations such as fibroblasts [40] and endothelial cells [41], in addition to ability to act upon themselves [42], is becoming clearer.

Here we show that cardiomyocytes alter their EV cargo when HCM sarcomeric mutations are present. Comparisons of mRNA transcripts within EVs released from HCM and WT identified “catalytic activity on a protein” and “protein binding” as the two functional pathways most affected by the presence of the disease mutation. The presence of calcium signaling genes, such as *CAMK2D*, *ATP2B4*, and *CAPN1*, is of particular note as abnormal calcium handling is common throughout HCM mutations. However, evidence suggests different HCM mutations lead to mutation-specific alterations in calcium handling [43], and as such the differences seen in our cells and their EV cargo may be specific to the c.*ACTC1*^G301A^ mutation.

Comparisons of either the HCM or the WT EVs and their parental cell lines showed that 65.1% of enriched transcripts are mutation dependent, showing the extent of the mutation effect on EV signaling. Pathway enrichment analysis of the EV mRNAs found to be mutation dependent, identified many potential signaling functions, with the greatest change observed in the cadherin signaling pathway (Fig. 3D).

Cadherins have previously been implicated in the regulation of hypertrophy with the modulation of cadherin-meditated adhesion inducing hypertrophy cardiomyocytes and linked to early onset HCM [44]. In addition, a study that profiled the mRNA cargo of serum-derived exosomes from patients with heart disease also identified altered cadherin signaling [45]. This same study also identified the presence of >200 differentially expressed lncRNAs within the serum-derived heart disease exosomes, with functions unknown.

One finding of particular note was the presence of differential levels of snoRNAs between WT and HCM hiPSC-CM EVs (Supplementary Fig. S5). As previously stated, studies have identified snoRNA involvement in cardiometabolic disease, including in lipid metabolism [14], diabetes mellitus [15], and doxorubicin cardiotoxicity [16]. Of interest, snoRNAs have also been shown to regulate splicing in normal cardiac development [17] and can alter splicing in some congenital heart diseases (tetralogy of Fallot) [18]. The snoRNAs identified in this study as differentially regulated in HCM EVs were predicted to have roles in both PTMs and alternative splicing, offering a potential mechanistic link between aberrant signaling and the presentation of these processes in HCM.

Of note, multiple paralogs of SNORD116 were detected with increased abundance (>2fc) in HCM hiPSC-CM EVs compared with WT hiPSC-CM EVs (Supplementary Fig. S5). The SNORD116 locus is encoded on chromosome 15 as a ∼57,000 bp cluster consisting of 30 paralogs of SNORD116, each roughly 100 bp in length [46]. Although SNORD116 expression is detected at highest levels in brain tissue, it is also found at lower levels in other tissues including heart, ovary, prostate, thyroid, and kidney [47,48]. SNORD116 is a disease-associated snoRNA, with all deletions and mutations reported for Prader–Willi syndrome (PWS) resulting in a loss of expression of this cluster [49].

PWD is a complex multisystem disorder that is the most common cause of life-threatening obesity in humans [50]. A link between PWS and an increased prevalence of congenital defects has been established, with congenital heart defects being the most common of these [51]. This raises the interesting possibility that SNORD116 may contribute to congenital heart disease and exploring its role within HCM EVs merits further investigation.

Of the 10 snoRNAs with increased abundance in HCM hiPSC-CM EVs, one other apart from SNORD166 has previously been reported in human disease. SNORD43 is one of the four snoRNAs that has been reported as dysregulated in cancer [52]. Its potential roles in directing 2′-*O*-ribose methylation in ribosome biogenesis may contribute to disease signaling [13], although more investigation is needed to determine if this is contributing to the HCM phenotype. Although there is evidence that 2′-*O*-ribose methylation may contribute to heart disease, with a specific set of snoRNAs found at the 14q32 locus, guiding these RNA modifications in associated heart failure [53].

### Transcriptomic changes in HCM during increased work

Sudden death remains the most visible and damaging complication associated with HCM [54]. Intense exercise represents a major risk factor in sudden death and as such, young athletes with HCM are often prevented from strenuous competition [55]. In this study, we subjected HCM cardiomyocytes to 2 Hz electrical stimulation to model an increased contraction workload that commonly proceeds to sudden death. We observed that 2 Hz pacing stimulation altered the expression of 66 genes regardless of the disease mutation, with 40 of these identified within the macromolecule metabolic process (Fig. 4C).

The altered metabolic expression in response suggested that our model was successful in subjecting the cardiomyocytes to increased energy demand and they were responding accordingly. Of interest, the presence of the disease mutation has a substantial additive effect, altering the expression of a further 1,058 genes. This additive effect may be responsible for the abnormal HCM response to an energy depleted state. HCM cardiomyocytes responded to the increased workload with altered gene expression profile in processes involved in biogenesis and morphogenesis, cardiac and circulatory development, and cellular communication and signaling.

The altered cellular communication and signaling gene expression observed translated through to differences in EV signaling. Whereas WT hiPSC-CMs increased EV release rate by 1.2-fold during 2 Hz stimulation, HCM hiPSC-CMs had a greater response of 1.5-fold (*P* < 0.0001), suggesting a role for exosome signaling in the HCM energy depletion response.

Analysis of these 2 Hz stimulated exosomes revealed a notable difference in small RNA cargo during this HCM signaling response. Following 2 Hz stimulation, the small RNA cargo only changed within HCM hiPSC-CMs EVs and not within WT EVs. Although the identities of these snoRNAs differed from those identified at baseline contraction frequency, the functional roles were similarly predicted to regulate PTMs and alternative splicing. As such, the change in EV snoRNA signaling may indicate a role for PTMs and alternative splicing in HCM signaling at baseline and during a further energy depletion state.

In conclusion, our data support previous findings that HCM mutations disrupt normal metabolic activities, and this worsens disproportionally in response to increased workload. In addition, we found sarcomeric mutations caused differential splicing within known HCM pathways. Intriguingly, we observed differences in snoRNA cargo within HCM released EV signals, which specifically altered in HCM mutant and not WT cardiomyocyte EVs, when workload was increased. These snoRNAs were predicted to regulate PTMs and splicing, pathways previously suggested to be common throughout HCM mutants.

Therefore further investigation is warranted to determine whether snoRNAs play a common role in the convergence of HCM phenotypes. The therapeutic potential of RNA-targeting in cardiovascular disease is currently being explored in multiple clinical trials, including both antisense oligonucleotides and small interfering RNA therapies [56]. As such, the identification of snoRNAs as targets in the treatment common HCM phenotypes opens a large field of potential future therapies.

## Supplementary Material

Supplemental data

Supplemental data

Supplemental data

Supplemental data

Supplemental data
