# Supplementary figures and images for: Transcriptomic Analysis of Cardiomyocyte Extracellular Vesicles in Hypertrophic Cardiomyopathy Reveals Differential snoRNA Cargo
Source: Stem Cells Dev. 2021 Dec 16;30(24):1215–27. doi: 10.1089/scd.2021.0202 (PMC8742282; doi:10.1089/scd.2021.0202)

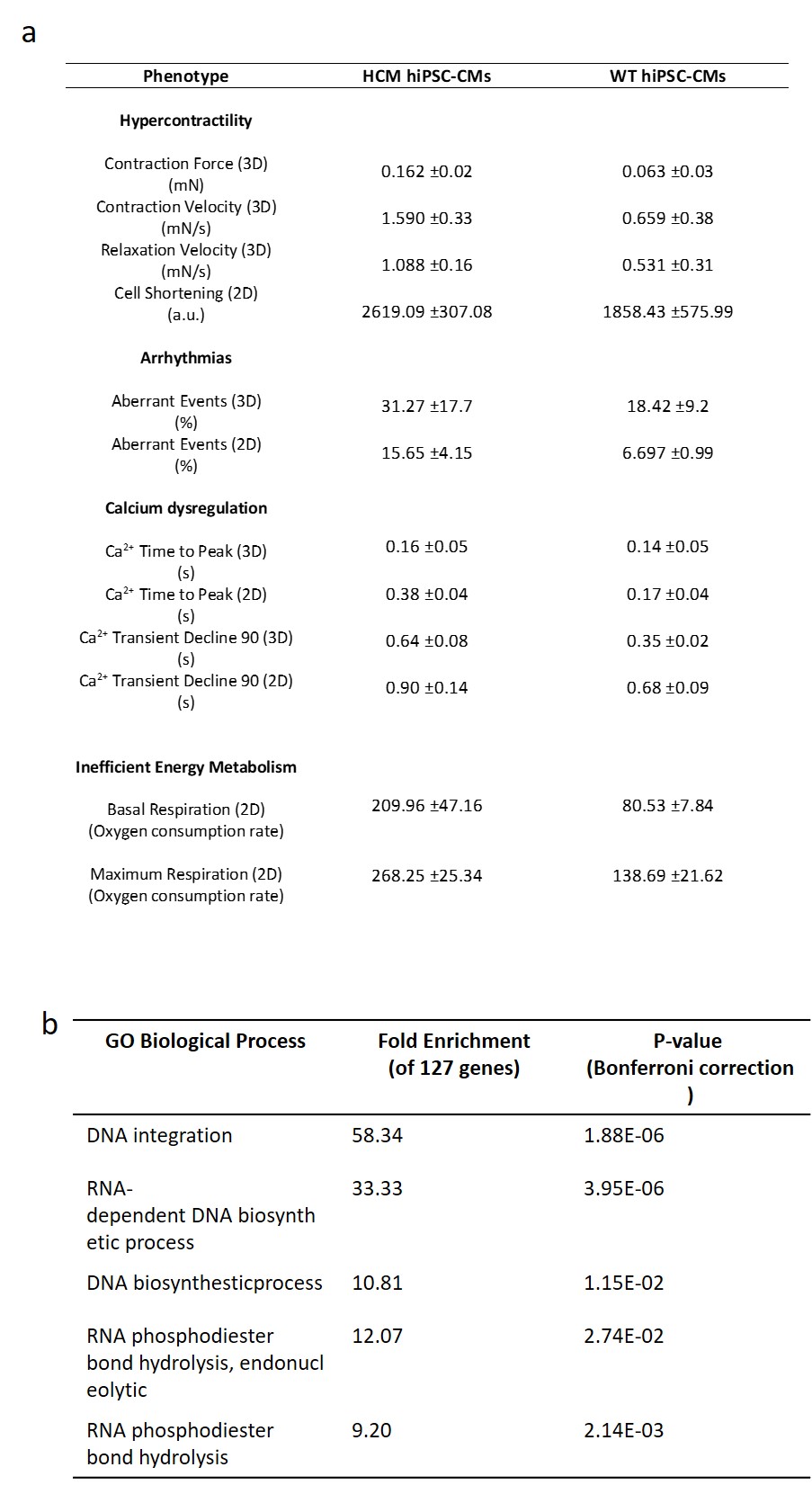

Supplement: Supplemental data [file Suppl_Fig1.jpg]

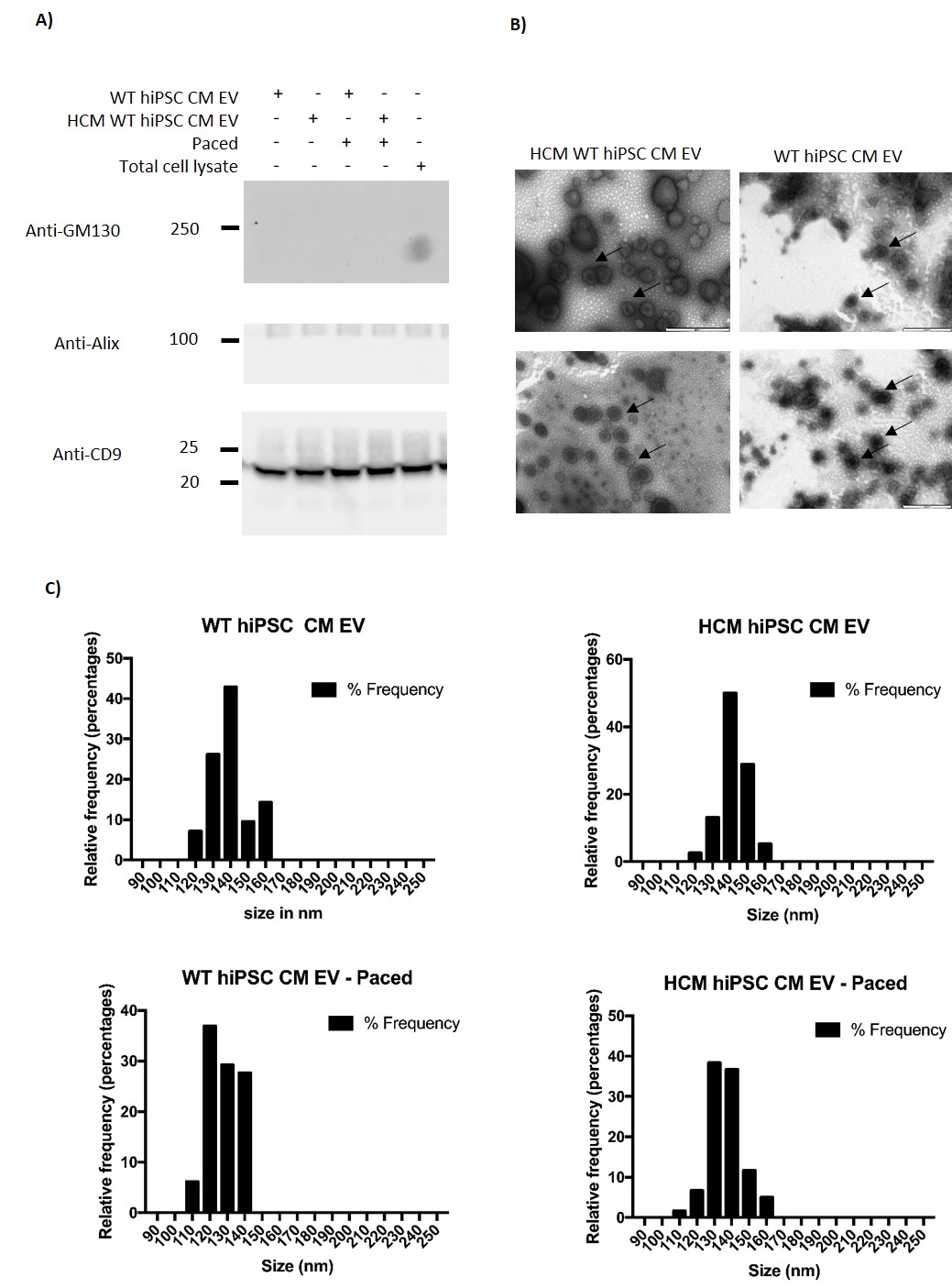

Supplement: Supplemental data [file Suppl_Fig2.jpg]

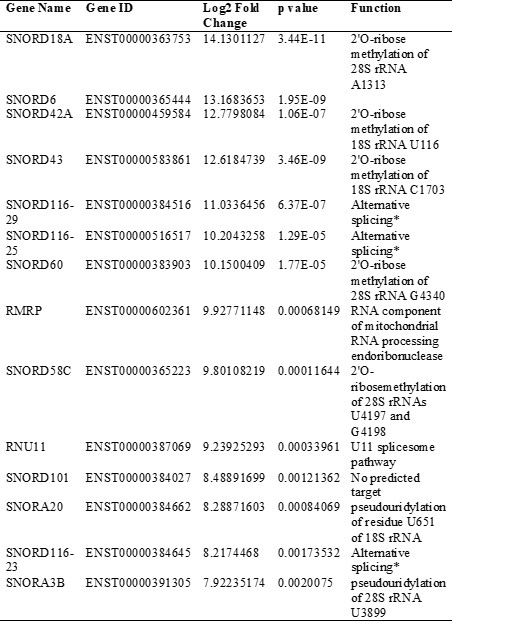

Supplement: Supplemental data [file Suppl_Fig5.jpg]

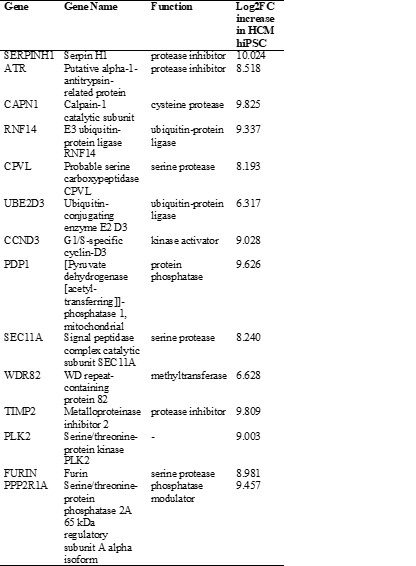

Supplement: Supplemental data [file Suppl_Fig3.jpg]

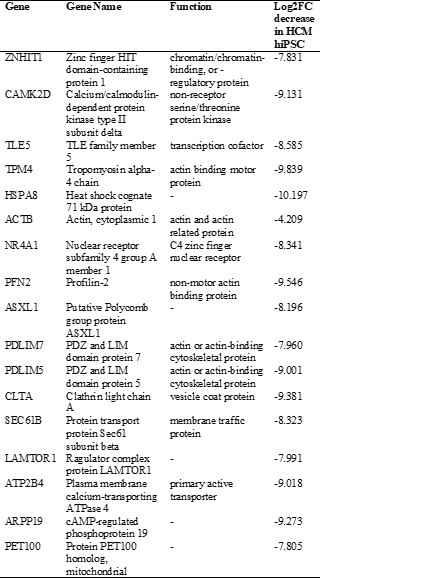

Supplement: Supplemental data [file Suppl_Fig4.jpg]
